# Cardiometabolic multimorbidity is associated with a worse Covid-19 prognosis than individual cardiometabolic risk factors: a multicentre retrospective study (CoViDiab II)

**DOI:** 10.1186/s12933-020-01140-2

**Published:** 2020-10-01

**Authors:** Ernesto Maddaloni, Luca D’Onofrio, Francesco Alessandri, Carmen Mignogna, Gaetano Leto, Giuseppe Pascarella, Ivano Mezzaroma, Miriam Lichtner, Paolo Pozzilli, Felice Eugenio Agrò, Monica Rocco, Francesco Pugliese, Andrea Lenzi, Rury R. Holman, Claudio Maria Mastroianni, Raffaella Buzzetti, the CoViDiab Study Group, Camilla Ajassa, Rugova Alban, Francesco Alessandri, Federica Alessi, Raissa Aronica, Valeria Belvisi, Raffaella Buzzetti, Matteo Candy, Alessandra Caputi, Anna Carrara, Elena Casali, Eugenio Nelson Cavallari, Giancarlo Ceccarelli, Luigi Celani, Maria Rosa Ciardi, Lucia Coraggio, Ambrogio Curtolo, Claudia D’Agostino, Gabriella D’Ettorre, Luca D’Onofrio, Francesca De Giorgi, Gabriella De Girolamo, Valeria Filippi, Lucio Gnessi, Cecilia Luordi, Ernesto Maddaloni, Claudio Maria Mastroianni, Ivano Mezzaroma, Carmen Mignogna, Chiara Moretti, Francesco Pugliese, Gregorio Recchia, Marco Ridolfi, Francesco Eugenio Romani, Gianluca Russo, Franco Ruberto, Giulia Savelloni, Guido Siccardi, Antonio Siena, Sara Sterpetti, Serena Valeri, Mauro Vera, Lorenzo Volpicelli, Mikiko Watanabe, Massimo Aiuti, Giuseppe Campagna, Cosmo Del Borgo, Laura Fondaco, Blerta Kertusha, Frida Leonetti, Gaetano Leto, Miriam Lichtner, Raffaella Marocco, Renato Masala, Paola Zuccalà, Felice Eugenio Agrò, Giulia Nonnis, Giuseppe Pascarella, Paolo Pozzilli, Alessandra Rigoli, Alessandro Strumia, Daniela Alampi, Monica Rocco

**Affiliations:** 1 Umberto I “Policlinico” General Hospital, Sapienza University of Rome, Viale Regina Elena 324, 00161 Rome, Italy; 2 Diabetes Trial Unit, Radcliffe Department of Medicine, University of Oxford, Oxford, UK; 3 Santa Maria Goretti Hospital, Polo Pontino Sapienza University, Latina, Italy; 4 Campus Bio-Medico University of Rome, Rome, Italy; 5 Sant’Andrea Hospital, Sapienza University of Rome, Rome, Italy

**Keywords:** Covid-19, Diabetes, SARS-CoV-2, Hypertension, COPD

## Abstract

**Background:**

Cardiometabolic disorders may worsen Covid-19 outcomes. We investigated features and Covid-19 outcomes for patients with or without diabetes, and with or without cardiometabolic multimorbidity.

**Methods:**

We collected and compared data retrospectively from patients hospitalized for Covid-19 with and without diabetes, and with and without cardiometabolic multimorbidity (defined as ≥ two of three risk factors of diabetes, hypertension or dyslipidaemia). Multivariate logistic regression was used to assess the risk of the primary composite outcome (any of mechanical ventilation, admission to an intensive care unit [ICU] or death) in patients with diabetes and in those with cardiometabolic multimorbidity, adjusting for confounders.

**Results:**

Of 354 patients enrolled, those with diabetes (n = 81), compared with those without diabetes (n = 273), had characteristics associated with the primary composite outcome that included older age, higher prevalence of hypertension and chronic obstructive pulmonary disease (COPD), higher levels of inflammatory markers and a lower PaO2/FIO2 ratio. The risk of the primary composite outcome in the 277 patients who completed the study as of May 15^th^, 2020, was higher in those with diabetes (Adjusted Odds Ratio (_adj_OR) 2.04, 95%CI 1.12–3.73, p = 0.020), hypertension (_adj_OR 2.31, 95%CI: 1.37–3.92, p = 0.002) and COPD (_adj_OR 2.67, 95%CI 1.23–5.80, p = 0.013). Patients with cardiometabolic multimorbidity were at higher risk compared to patients with no cardiometabolic conditions (_adj_OR 3.19 95%CI 1.61–6.34, p = 0.001). The risk for patients with a single cardiometabolic risk factor did not differ with that for patients with no cardiometabolic risk factors (_adj_OR 1.66, 0.90–3.06, _adj_p = 0.10).

**Conclusions:**

Patients with diabetes hospitalized for Covid-19 present with high-risk features. They are at increased risk of adverse outcomes, likely because diabetes clusters with other cardiometabolic conditions.

## Background

Cardiometabolic disorders have been described among the most important chronic underlying conditions worsening Coronavirus disease 2019 (Covid-19) outcomes [1–5], with hypertension and type 2 diabetes being frequent comorbidities in patients with Covid-19 who require intensive care or die [6, 7]. Type 2 diabetes, in particular, might hypothetically impact on all of the different aspects of SARS-CoV-2 infection, from the contagion to the clinical presentation and to disease severity [8]. Further, in-hospital hyperglycaemia has been associated with worse Covid-19 outcomes [9], being a negative prognostic factor at hospital admission in both patients with and without diabetes [10]. In addition, regardless of diabetes diagnosis, hyperglycaemia reduced the efficacy of treatment with Tocilizumab in patients affected by Covid-19 [11]. On the other hand, a recent report from Wuhan failed to show an independent association of type 2 diabetes with Covid-19 mortality after adjustment for other cardiovascular conditions [12]. Notably, most cardiometabolic disorders share a common pathogenic soil, often cluster together, and might reflect the same intermediate pathways that favour Covid-19 progression [13–15]. Therefore, assessing the possible association of type 2 diabetes with Covid-19 outcomes based on individual cardiometabolic disorders may be subject to a collider bias, leading to distorted results [16, 17]. This makes it difficult to disentangle independent associations Covid-19 may have with single components of cardiometabolic multimorbidity, defined here as a group of main metabolic disorders that increase the risk of cardiovascular events, such as diabetes, hypertension and dyslipidaemia. Nevertheless, most studies investigating diabetes as a risk factor for Covid-19 progression searched for independent associations, leading to conflicting conclusions [12, 18–20]. We, instead, hypothesized that people with diabetes may differ from those without diabetes in their clinical presentation, course and prognosis of Covid-19 due to the propensity of diabetes to cluster with other cardiometabolic risk factors, such as hypertension and/or dyslipidaemia, which contribute to the increased pro-inflammatory and hypercoagulable states of people with diabetes. Furthermore, most studies published to date on this topic come from Asian countries, with few data available from Western countries where differences in ethnic groups and healthcare systems may lead to different associations [21, 22].

Overall, there is an urgent need for additional data to clarify the relationships between diabetes, cardiometabolic multimorbidity and Covid-19 [18] which could provide significant insights of global health interest to help tackle this deadly pandemic in a large group of at-risk individuals. We aimed to describe in detail, using opportunistic data collected retrospectively, the clinical and biochemical features of patients with and without diabetes hospitalized for Covid-19 in four academic hospitals in the Lazio region, Italy, to evaluate their outcomes, and to evaluate the impact of cardiometabolic multimorbidity.

## Materials and methods

### Study design and population

The Covid-19 & Diabetes (CoViDiab) study is a multi-center observational study which collected data retrospectively from medical charts of patients hospitalized for Covid-19 from March 1^st^ to May 15^th^, 2020 in four academic hospitals located in the Lazio region of Italy: Umberto I “Policlinico” General hospital and Sant’Andrea hospital, Sapienza University of Rome; Santa Maria Goretti hospital, Polo Pontino of Sapienza University in Latina; Campus Bio-Medico University hospital in Rome [23]. Patients eligible for inclusion were aged ≥ 18 years old with a diagnosis of Covid-19 confirmed by at least one real-time polymerase chain reaction assay, in accordance with the protocol established by the World Health Organisation [24]. After exclusion of 19 patients with unknown diabetes status, baseline data for 354 patients and clinical outcomes for 277 patients up to May 15^th^, 2020 were available for inclusion in this analysis.

### Study outcomes

The CoViDiab primary aim was to evaluate whether patients with diabetes, compared with those without diabetes, were at increased risk of adverse Covid-19 outcomes, independent of age and sex. The composite primary outcome was defined as any of mechanical ventilation, admission to an intensive care unit (ICU), or death. Pre-specified secondary endpoints included a composite outcome of ICU admission or death and all-cause mortality (ACM). In this study, we did not seek to test whether diabetes as a risk factor for Covid-19 progression is independent of hypertension and dyslipidaemia, which may be considered as coexisting components of a single cardiometabolic disorder (or syndrome). Instead, our secondary aim (if diabetes was confirmed to be associated with an increased risk of the primary composite outcome) was to evaluate whether cardiometabolic multimorbidity (defined as ≥ 2 of three risk factors of diabetes, hypertension and dyslipidaemia) may be considered as a risk factor that differs from a single cardiometabolic condition. Accordingly, patients were stratified into three mutually exclusive cardiometabolic groups: no conditions, one condition and two or three conditions.

### Data collection strategy and definitions

Data collected included: demographic information (age and sex); presence of diabetes (defined as at least one random blood glucose value > 200 mg/dl, or fasting blood glucose > 126 mg/dl, or HbA_1c_ > 6.5%, or self-reported history of diabetes with ongoing anti-diabetes therapy), type of diabetes (type 1, type 2, other); smoking habits (never, ex, current); prior history of hypertension, dyslipidemia, chronic obstructive pulmonary disease (COPD), heart failure, cardiovascular events (myocardial infarction, percutaneous coronary intervention, coronary artery-bass graft or stroke), malignancy (any neoplasia diagnosed within the last five years or active neoplasia); presenting symptoms of SARS-CoV-2 infection (fever, cough, cold, conjunctivitis, chest pain, dyspnea, nausea, vomiting, diarrhea). Biochemical data measured at admission, where available, were: plasma glucose, serum creatinine, erythrocyte sedimentation rate (ESR), C-reactive protein (CRP), full blood count, lactate dehydrogenase, fibrinogen, D-dimer and blood gas analysis. Body mass index (BMI) was calculated for the 169 patients with height and weight data available. Usual care medications at admission were ascertained from those reported by the inpatient-accepting physician. Diabetes usual care medications were also retrieved from the web-based reimbursement system of Lazio region (WebCare Lazio), as categorized by this system: euglycaemic agents (EuGlA: metformin, dipeptidyl peptidase 4 inhibitors [DPP4i], glucagon-like peptide 1 receptor agonists [GLP-1RA], sodium-glucose co-transporter 2 inhibitors [SGLT2i] and/or pioglitazone); oral hypoglycaemic agents (OHA: sulfonylureas or glinides); basal insulin (alone or in combination with EuGlA or OHA); multiple daily insulin injections (MDI: ≥ 3 insulin injections per day). The WebCare Lazio system was also used to confirm a self-reported history of diabetes.

### Statistical analysis

Continuous variables are presented as medians [25th–75th percentile]. Categorical variables are presented as number and percentages, calculated on the data available. We made no assumptions regarding missing data. Kruskal–Wallis, Chi-squared and Fisher exact tests were used for comparisons between groups, as appropriate.

We estimated that at least 200 patients completing the study would be required to provide 80% power to detect a 2.5-fold higher incidence of the primary composite outcome in patients with diabetes hospitalized for Covid-19, compared with those without diabetes, using a one-sided alpha-level of 0.05 and allowing for adjustment of sex and age.

Logistic regression models adjusted for age and sex were used to investigate associations of the primary and secondary outcomes with diabetes, and with other risk factors explored in the study, namely hypertension, dyslipidemia, COPD, heart failure, previous cardiovascular events, malignancy and smoking status (never *vs.* ever). The secondary aim of the study (association of cardiometabolic multimorbidity with the primary composite outcome) was also explored using a logistic regression model adjusted for age, sex and risk factors (other than hypertension, diabetes and dyslipidemia) that were univariately associated (p < 0.1) with the outcome after correction for age and sex. The Wald test was used to test equality of the regression coefficients between cardiometabolic groups. Stata/IC 12.1 software was used for data analysis and Prism 8.4 Software for graphical presentations.

### Ethics

CoViDiab complies with the principle of the Helsinki Declaration and was approved by the Ethical Committee of Umberto I “Policlinico” General hospital. Because of the study’s retrospective design, informed consent was waived for patients who had been discharged, could not be contacted, or died. The privacy and anonymity of the data collected was guaranteed in accordance with current regulations.

## Results

### Clinical and biochemical features of Covid-19 patients with concomitant diabetes

Presenting characteristics for all 354 patients are listed in Table 1. Patients with diabetes, compared with those without diabetes, were older (age ≥ 70 years: 64.2% *vs.* 33.0%, p < 0.001) but with a similar sex distribution. They also presented with higher rates of hypertension (66.7% *vs.* 46.1%, p = 0.001), dyslipidemia (37.2% *vs.* 18.0%, p < 0.001), prior cardiovascular events (16.0% *vs.* 7.7%, p = 0.026), heart failure (12.8% *vs.* 4.1%, p = 0.005) and COPD (21.2% *vs.* 10.0%, p = 0.008). No differences in smoking habits or malignancy rate were found. BMI did not differ between patients with and without diabetes.

**Table 1:** Baseline features of hospitalized Covid-19 patients with diabetes compared with those without diabetes, and patients experiencing the primary composite outcome (admission to ICU, mechanical ventilation or death), compared with those discharged alive not experiencing an event

	Diabetes			Primary composite outcome		
	No n = 273	Yes n = 81	p	No n = 149	Yes n = 128	p
Age ≥ 70, n (%)	90 (33.0)	52 (64.2)	< 0.001	42 (28.2)	65 (50.8)	< 0.001
Male sex, n (%)	165 (60.2)	50 (61.7)	0.81	84 (56.4)	86 (67.2)	0.065
Body mass index (Kg/m^2^)	26.1 [23.9–28.6]*	27.0 [24.8–29.4]**	0.20	25.2 [23.4–27.7]***	27.1 [25.0–29.4]****	0.002
Diabetes, n (%)	N/A	N/A	N/A	23 (15.4)	40 (31.2)	0.002
Diabetes therapy, n (%)	N/A	N/A	N/A			
Diet alone, n (%)				1 (4.8)	4 (10.8)	0.64
EuGla, n (%)				9 (42.9)	17 (45.9)	0.82
OHA (alone or in combination with EuGlA), n (%)				4 (19.0)	3 (8.1)	0.22
Basal insulin (alone or in combination with EuGlA or OHA), n (%)				0 (0.0)	4 (10.8)	0.29
MDI, n (%)				7 (33.3)	9 (24.3)	0.46
Hypertension, n (%)	126 (46.1)	54 (66.7)	0.001	62 (41.6)	86 (67.2)	< 0.001
Drugs acting on RAAS						
ACEi, n (%)	− 36 (13.2)	− 19 (23.5)	0.011	24 (16.1)	23 (18.0)	0.55
ARBs, n (%)	− 40 (14.7)	− 14 (17.3)	0.42	20 (13.4)	19 (14.8)	0.60
Dyslipidaemia, n (%)	49 (18.0)	29 (37.2)	< 0.001	28 (18.9)	37 (29.1)	0.047
Smoking	^	^^		^^^	^^^^	
Never, n (%)	203 (83.2)	55 (75.3)	0.13	100 (77.5)	92 (80.7)	0.54
Ever, n (%)	41 (16.8)	18 (24.6)		29 (22.9)	22 (19.3)	
Ex, n (%)	31 (12.7)	17 (23.3)	0.055^#^	19 (14.7)	21 (18.4)	0.032^#^
Current, n (%)	10 (4.1)	1 (1.4)		10 (7.8)	1 (0.9)	
Prior CV event, n (%)	21 (7.7)	13 (16.0)	0.026	14 (9.5)	16 (12.5)	0.42
Prior heart failure, n (%)	11 (4.1)	10 (12.8)	0.005	6 (4.1)	13 (10.6)	0.038
Prior malignancy, n (%)	14 (5.2)	6 (7.7)	0.40	6 (4.0)	10 (7.9)	0.17
Prior COPD, n (%)	27 (10.0)	17 (21.2)	0.008	11 (7.4)	28 (21.9)	0.001
Antiviral therapy, n (%)	103 (37.7)	29 (35.8)	0.77	60 (40.3)	60 (46.9)	0.27
Plasma glucose (mg/dL)	102 [93–118]	151 [117–215]	< 0.001	101 [93–119]	119 [99–160]	< 0.001
Serum creatinine (mg/dL)	0.81 [0.70–1.04]	1.03 [0.82–1.50]	< 0.001	0.83 [0.70–1.03]	0.97 [0.72–1.26]	0.016
ESR (mm/hr)	42.5 [23.5–59]	73.5 [43–86]	0.075	38 [24–65]	78 [54–79]	0.19
CRP (mg/L)	2.71 [0.82–7.52]	5.06 [1.70–10.58]	0.014	1.84 [0.59–4.17]	6.37 [2.09–13.52]	< 0.001
Lactate dehydrogenase (U/L)	272.5 [216–344]	289 [218–386]	0.31	254 [210–314]	313 [246–399]	< 0.001
Fibrinogen (mg/dL)	470.5 [383.5–800]	556 [467–752]	0.028	479 [372–800]	524 [426–800]	0.064
D-dimer (mg/L)	450 [1.57–891]	432 [4.09–1440]	0.31	357 [0.89–780]	598 [214–1250]	0.006
White blood cell count (× 10^9^/L)	5.90 [4.38–8.48]	6.79 [5.46–8.80]	0.045	5.73 [4.08–7.20]	7.04 [5.11–9.67]	< 0.001
Lymphocytes (× 10^9^/L)	1.11 [0.73–1.66]	1.07 [0.75–1.48]	0.38	1.20 [0.81–1.65]	0.95 [0.65–1.52]	0.015
Neutrophil count (× 10^9^/L)	3.97 [2.67–6.0]	4.84 [0.75–1.48]	0.017	3.78 [2.62–5.12]	5.25 [3.65–7.7]	< 0.001
Blood gas analysis						
pH	7.46 [7.42–7.49]	7.46 [7.42–7.48]	0.45	7.45 [7.43–7.48]	7.46 [7.41–7.50]	0.95
PaO_2_/FIO_2_ ratio	367 [281–438]	322 [237–394]	0.012	405 [350–462]	281 [186–333]	< 0.001
pCO_2_, mmHg	34.0 [31.0–37.0]	35.7 [33.0–39.0]	0.055	34.9 [31.0–37.0]	35 [31.0–38.2]	0.43
HCO_3_, mmol/l	24.2 [22.2–26.0]	24.6 [22.4–27.1]	0.45	24.0 [22.4–25.5]	24.5 [22.0–28.0]	0.16
Venous lactate, mmol/l	1.2 [0.9–2.1]	1.7 [1.2–7.0]	< 0.001	1.2 [0.8–1.8]	1.5 [1.0–8.0]	0.002

Continuous variables are presented as median [25th, 75th percentile]; categorical variables are presented as number (percentage)

^#^ p-value for difference in never, ex and current mokers

EuGlA, euglycemic agents (metformin, dipeptidyl peptidase 4 inhibitors, glucagon-like peptide 1 receptor agonists, sodium-glucose co-transporter 2 inhibitors and/or pioglitazone); OHA, oral hypoglycaemic agents (sulfonylureas or glinides); MDI, multiple daily insulin injections; RAAS, renin–angiotensin–aldosterone system; ACEi, angiotensin converting enzyme inhibitors; ARB, angiotensin receptor blocker; CV, cardiovascular; COPD, chronic obstructive pulmonary disease; ESR, erythrocytes sedimentation rate; CRP, c-reactive protein; PaO2, arterial pO2; FIO2, fraction of inspired oxygen

Body mass index data were available for *120, **49, ***55 and ****95 patients

Smoking data were available for ^244, ^^73, ^^^129 and ^^^^114 patients

Anti-diabetes therapy data were available for 58 of the 63 patients with diabetes who completed the study

At hospital admission patients with diabetes, compared with those without diabetes, presented with higher values of plasma glucose (151 [117–215] *vs.* 102 [93–118] mg/dL, < 0.001), serum creatinine (1.03 [0.82–1.50] *vs.* 0.81 [0.70–1.04] mg/dL, p < 0.001), CRP (5.06 [1.70-–10.58] *vs.* 2.71 [0.82–7.52] mg/L, p = 0.014), fibrinogen (556 [467–752] *vs.* 471 [384–800] mg/dL, p = 0.028), white blood cell count (6.79 [5.46–8.80] *vs.* 5.90 [4.38–8.48] × 10^9^/L, p = 0.045) and neutrophil count (4.84 [0.75–1.48] *vs.* 3.97 [2.67–6.0] × 10^9^/L, p = 0.017). Those with diabetes also had a lower median PaO_2_/FIO_2_ ratio (322 [237–394] *vs.* 367 [281–438], p = 0.012) and a higher venous lactate (1.8 [1.2–7.0] *vs.*1.2 [0.9–2.1], p < 0.001).

No differences were observed in the frequencies of SARS-CoV-2 infection presenting symptoms between patients with and without diabetes (Additional file 1: Table S1).

### Primary composite outcome

The primary composite outcome occurred in 128 (46.2%) of the 277 patients who completed the study (discharged alive or experiencing at least one component of the primary composite outcome). Differences in clinical features between those experiencing the primary composite outcome and those discharged alive (n = 149), mostly mirrored the differences seen between patients with and without diabetes (Table 1). Those with, compared with those without the primary composite outcome, were more often > 70 years old (p < 0.001) and more likely to have hypertension (p < 0.001), dyslipidemia (p = 0.047), heart failure (0.038) or COPD (p = 0.001). There was no difference in prior history of CV events between groups but those with the primary composite outcome were less often current smokers than never smokers (0.9% *vs.* 7.8%, p = 0.032). BMI in the 150 patients with BMI data and complete follow-up, was higher in those with the primary composite outcome compared with those discharged alive not requiring neither ICU admission or mechanical ventilation (27.1 [25.0–29.4] vs 25.2 [23.4–27.7] Kg/m^2^, p = 0.002).

Differences in biochemical features between patients with, compared with those without the primary composite outcome, also paralleled the differences seen between patients with and without diabetes (Table 1). Those experiencing the primary composite outcome had higher plasma glucose (p < 0.001), serum creatinine (p = 0.016), CRP (p < 0.001), white blood cell count (p < 0.001), neutrophil count (p < 0.001) and venous lactate (p = 0.002), and a lower PaO_2_/FIO_2_ ratio (p < 0.001). They also had higher lactate dehydrogenase (p < 0.001) and D-dimer (p = 0.006) concentrations, but had a lower lymphocyte count (p = 0.015). Frequencies of SARS-CoV-2 infection presenting symptoms were similar among patients with, compared with those without the primary composite outcome, apart from dyspnea which was more frequent among those experiencing the primary composite outcome (66.1% *vs.* 40.9%, p < 0.001) (Additional file 1: Table S1).

**Fig. 1:**
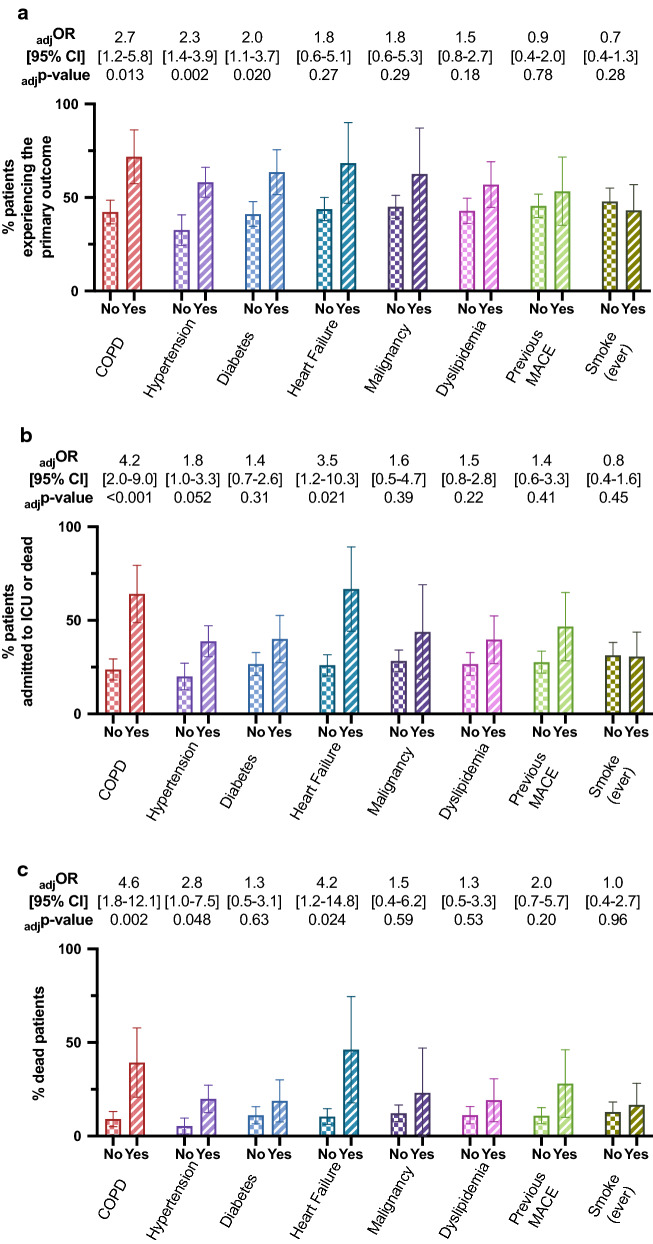
Proportion of patients experiencing the primary composite outcome (**a**), and the secondary outcomes of ICU admission or death (**b**), or death (**c**) among patients with or without different comorbidities. Age and sex adjusted odds ratios (OR) with 95% confidence intervals (CI) for those with, compared with those without, each comorbidity are reported. Error bars represent 95% confidence intervals

Age and sex adjusted regression models confirmed that Covid-19 patients with the primary composite outcome were more likely to have diabetes (adjusted odds ratio [_adj_OR] 2.04, 95% confidence interval [CI] 1.12–3.73, p = 0.020), hypertension (_adj_OR 2.31, 95%CI 1.37–3.92, p = 0.002) or COPD (_adj_OR 2.67, 95%CI 1.23–5.80, p = 0.013), while the associations with dyslipidemia and heart failure were lost (Fig. 1a).

Therapies for diabetes at admission did not differ between patients with diabetes in whom the primary composite outcome did or did not occur (Table 1). Similarly, there was no difference in the use of angiotensin-converting enzyme (ACE) inhibitors or angiotensin receptor blockers (ARB) between patients who did or did not experience the primary outcome (18.0% vs 16.1%, p = 0.55 and 14.8% vs 13.4%, p = 0.60, respectively).

### Relationship between cardiometabolic multimorbidity and the primary composite outcome

Of the 277 patients completing the study, 100 (36.1%) did not have diabetes, hypertension or dyslipidaemia, 96 (34.7%) had just one of these risk factors (16 with diabetes, 70 with hypertension, 10 with dyslipidaemia), and 81 (29.2%) had ≥ two of these risk factors meeting our study definition of cardiometabolic multimorbidity. The proportion of patients > 70 years old (p < 0.001), with a prior cardiovascular event (p < 0.001), heart failure (p = 0.007) or COPD (p = 0.002), and higher concentrations of plasma glucose (p < 0.001), creatinine (p = 0.010), CRP (p = 0.043) and venous lactate (p < 0.001) increased with increasing numbers of cardiometabolic conditions, and with a decreasing PaO_2_/FIO_2_ ratio (p = 0.002) (Table 2).

**Table 2:** Patient baseline characteristics according to their number of cardiometabolic conditions (diabetes, hypertension, dyslipidaemia)

	Number of cardiometabolic conditions			
	None n = 100	One n = 96	Two or three n = 81	p
Age ≥ 70 years, n (%)	16 (16.0)	39 (40.6)	52 (64.2)	< 0.001
Male sex, n (%)	62 (62.0)	58 (60.4)	50 (61.7)	0.97
Body mass index (kg/m^2^)	25.4 [23.4–27.8]*	26.2 [24.6–28.7]**	27.2 [24.9–29.4]***	0.12
Smoking	^	^^	^^^	
Never, n (%)	73 (82.95)	66 (77.65)	53 (75.71)	0.57
Ever, n (%)	15 (17.05)	19 (22.35)	17 (24.29)	
Ex, n (%)	11 (12.5)	14 (16.47)	15 (21.43)	0.50^#^
Current, n (%)	4 (4.55)	5 (5.88)	2 (2.86)	
Prior CV event, n (%)	3 (3.0)	8 (8.33)	19 (23.75)	< 0.001
Prior heart failure, n (%)	3 (3.0)	5 (5.21)	11 (14.86)	0.007
Prior malignancy, n (%)	4 (4.0)	5 (5.21)	7 (8.97)	0.35
Prior COPD, n (%)	7 (7.0)	12 (12.5)	20 (25.0)	0.002
Plasma glucose (mg/dL)	100 [90–114]	112 [96–136]	119 [100–159.5]	< 0.001
Serum creatinine (mg/dL)	0.8 [0.7–1]	0.88 [0.7–1.04]	1.02 [0.74–1.50]	0.010
ESR (mm/hr)	44.5 [36–55]	24 [22–54]	78 [41–93]	0.10
CRP (mg/L)	2.29 [0.72–4.83]	3.18 [0.99–9.64]	4.58 [1.68–9.43]	0.043
Lactate dehydrogenase (U/L)	268 [216–318]	273 [211–370]	287 [223–382]	0.39
Fibrinogen (mg/dL)	501.5 [404–800]	464 [361–707]	538 [417–738]	0.46
D-dimer (mg/L)	416 [0.89–761.5]	464 [13.2–1250]	446 [2.2–1410]	0.23
White blood cell count (× 10^9^/L)	5.95 [4–8.48]	6.09 [5.09–8.84]	6.47 [4.64–8.21]	0.62
Lymphocyte count (× 10^9^/L)	1.11 [0.73–1.69]	1.07 [0.68–1.66]	1.08 [0.78–1.58]	0.81
Neutrophil count (× 10^9^/L)	3.97 [2.60–5.75]	4.44 [3.14–6.52]	4.33 [2.80.6.77]	0.34
Blood gas analysis				
pH	7.46 [7.43–7.49]	7.46 [7.42–7.49]	7.45 [7.42–7.49]	0.70
PaO_2_/FIO_2_ ratio	381 [319–462]	350 [269–419]	319 [240–398]	0.002
pCO_2_, mmHg	34 [31–37]	35 [32–39]	35.2 [31–38]	0.90
HCO_3_, mmol/l	24.2 [22.5–25.9]	24.7` [22.4–26.0]	24.0 [21.6–27.1]	0.33
Venous lactate, mmol/l	1.1 [0.8–1.6]	1.4 [0.9–6]	1.6 [1.1–7]	< 0.001

^#^p-value for differences in never, ex and current smokers

CV, cardiovascular; COPD, chronic obstructive pulmonary disease; ESR, erythrocytes sedimentation rate; CRP, c-reactive protein; PaO_2_, arterial pO_2_; FIO_2_, fraction of inspired oxygen. Body mass index data available for *45, **54 and ***51 patients

Smoking data available for ^88, ^^85 and ^^^70 patients

**Fig. 2:**
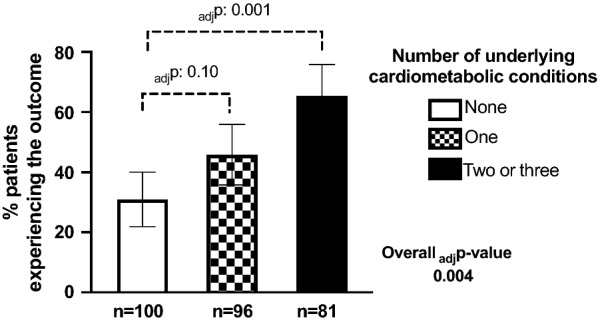
Proportion of patients experiencing the primary composite outcome according to their number of underlying cardiometabolic conditions (hypertension, diabetes, dyslipidaemia). P-values are adjusted for age, sex and COPD. Error bars represent 95% confidence intervals

The proportion of patients experiencing the composite primary outcome increased with increasing numbers of cardiometabolic risk factors (Fig. 2), independently of age, sex and COPD (_adj_p = 0.004). The risk of the primary composite outcome in patients with cardiometabolic multimorbidity, compared with those with no cardiometabolic risk factors, was higher (_adj_OR [95% CI] 3.19 [1.61–6.34], _adj_p = 0.001). They also were at higher risk when compared with patients with a single cardiometabolic risk factor (_adj_OR 1.92, 1.02–3.64, _adj_p = 0.045). The risk for patients with a single cardiometabolic risk factor, however, did not differ with that for those with no cardiometabolic risk factors (_adj_OR 1.66, 0.90–3.06, _adj_p = 0.10).

### Secondary outcomes

Analyses examining the secondary outcomes of ICU admission or death, and ACM alone, were performed for patients who were admitted to ICU, died or were discharged alive without ICU admission (n = 259), and for the those who died or were discharged alive at study end (n = 228). ICU admission or death occurred in 77 (29.7%) in the former, and 29 (12.7%) of the latter died. After adjustment for age and sex, diabetes was not associated with either of these secondary outcomes, whereas a prior history of COPD or heart failure was (Fig. 1b, c and Additional file 1: Table S2).

## Discussion

### Main findings

Our study shows that the characteristics associated with worse Covid-19 outcomes are found more frequently in patients with diabetes than in those without diabetes. These include older age, higher prevalence of chronic comorbidities such as hypertension or COPD, higher levels of inflammatory markers, and a lower PaO_2_/FIO_2_ ratio. Accordingly, the risk of progression towards mechanical ventilation, ICU admission or death was significantly higher among patients with diabetes than in those without, independent of age and sex. As we expected, only a minority of patients with diabetes (25.4%) had neither hypertension nor dyslipidemia, supporting our choice not to consider these risk factors as independent variables. This observation suggests that findings from studies reporting diabetes is not associated with Covid-19 severity after adjustment for other cardiovascular conditions should be interpreted with caution [12]. In line with our hypothesis, patients with cardiometabolic multimorbidity had a higher risk of the primary outcome compared with patients with no or a single cardiometabolic risk factor (diabetes, hypertension or dyslipidaemia). Compared with patients with no cardiometabolic risk factors, the primary composite outcome was also higher among patients with a single risk factor, but was not significant after adjustment for age, sex and presence of COPD. Of note, while drugs targeting the incretin system or the renin–angiotensin–aldosterone system have been hypothesized to be associated with Covid-19 outcomes [25, 26], their use did not differ between patients with or without the primary outcome.

### Covid-19 and cardiometabolic multimorbidity

Overall our results confirm previous findings from other countries that Covid-19 patients with diabetes are more likely to require intensive care or to die, compared with Covid-19 patients without diabetes [12, 19, 20, 27], and in addition suggest this association is driven by the presence of cardiometabolic multimorbidity rather than by diabetes alone. In this regard, categorizing patients as having cardiometabolic multimorbidity, rather than a simply summing risk factors [15], seems to almost completely explain the interaction between cardiometabolic disorders and Covid-19. Coexisting cardiometabolic risk factors may indeed either be the expression of a common pathogenic soil or cooperate with each other to predispose Covid-19 patients to progress towards more severe clinical scenarios. In patients presenting with diabetes but not hypertension or dyslipidemia, pathogenic pathways involved may not be sufficiently affected to impact on the clinical course of Covid-19.

The observation that cardiometabolic multimorbidity worsens Covid-19 is of clinical relevance, highlighting the importance of tackling cardiovascular risk as a whole to improve Covid-19 outcomes. Of note, an estimation of the overall effects of the Covid-19 outbreak according to underlying conditions has also suggested that cardiovascular comorbidities, together with COPD, may be responsible for the majority of excess deaths associated with Covid-19 pandemic from both direct and indirect effects [28].

### Hypothesized mechanisms underlying the association of cardiometabolic multimorbidity with Covid-19

Different mechanisms may be hypothesized to explain the association of cardiometabolic health with Covid-19 outcomes. It has been suggested that Covid-19 not only affects the respiratory system but also the vasculature [29–32]. Direct SARS-CoV-2 infection of endothelial cells causing endothelitis in several organs has been demonstrated in patients dying from Covid-19 [33], suggesting Covid-19 is an infectious disease affecting endothelial function. It is worth hypothesizing therefore, that cardiometabolic multimorbidity may predispose to worse Covid-19 outcomes by weakening endothelial cells [34], which then become more susceptible to viral infection. Additionally, the hypercoagulable and pro-inflammatory states often observed in cardiometabolic patients [35] may also contribute towards the formation of the multiple blood clots and the cytokine storm that can occur in the most severe Covid-19 cases [36, 37]. This endothelial hypothesis accords with recent data suggesting that a high amount of visceral adiposity, a common feature of cardiometabolic patients associated with chronic low-grade inflammation, associates with worse Covid-19 outcomes [38]. While measures of visceral adiposity were not available in our study, BMI was found to be higher in patients with the primary outcome, consistent with previous reports in other populations [4, 39]. The trend we noted towards higher BMI with increasing number of cardiometabolic risk factor in the relatively low number of patients with BMI data available our population was not statistically significant.

### Strengths and limitations

Limitations to our study include retrospective collection of data from electronic and paper records, relatively few patients with BMI data available, and incomplete follow-up of some patients without an endpoint who were still hospitalized at the time of this analysis. Also, we were not able to retrieve glycemic control data during hospitalization, which has been associated with worse Covid-19 outcomes [9, 20]. The small number of deaths does not allow us to make any conclusions about the non-significant association of diabetes with ACM, which was however associated with prior history of hypertension, COPD or heart failure. Finally, we were not able to estimate insulin resistance, or surrogates such as triglyceride-glucose index [40], in our population, which is often considered the common soil for cardiometabolic conditions. Similarly, the absence of waist circumference data not allow us to identify patients with the metabolic syndrome to assess its possible impact, although the utility of this categorisation in type 2 diabetes has been increasingly questioned [41]. Unfortunately, due to the observational study design and the demanding work condition determined by the pandemic, we were unable to collect additional blood samples or to perform additional radiological investigations to test bio-markers not routinely measured in all patients, such as cardiac troponin, interleukins, or to assess visceral adiposity, all of which may be involved in the relationship between cardiometabolic multimorbidity and Covid-19 progression [38, 42, 43]. Novel studies should be performed to evaluate whether the increased risk conferred by cardiovascular multimorbidity is associated to augmented cytokine storm and to central obesity.

Strengths of our study include a detailed characterization of the clinical and biochemical features of patients hospitalized for Covid-19, with and without diabetes, with good generalizability of the results thanks to the multicentre study design. Furthermore, to the best of our knowledge, this is the first study assessing Covid-19 outcomes in the context of cardiometabolic multimorbidity.

## Conclusion

Our study shows that patients with diabetes hospitalized for Covid-19 present with high-risk clinical and biochemical features and are at increased risk of mechanical ventilation, ICU admission or death, likely because diabetes frequently clusters with cardiometabolic multimorbidity.

## Supplementary information

**Additional file 1:Table S1.** SARS-CoV-2 infection symptoms at hospitalization in patients with, compared with those without, diabetes and in patients experiencing the primary composite compared with those without. **Table S2.** Odds ratio (OR) with [95% confidence intervals, CI] for secondary outcomes, unadjusted and adjusted for age and sex. Abbreviations: CV, cardiovascular; COPD, chronic obstructive pulmonary disease.

## Data Availability

The datasets used and/or analysed during the current study are available from the corresponding author on reasonable request.

## Abbreviations

ACE: Angiotensin converting enzyme
ACM: All-cause mortality
ARB: Angiotensin receptor blocker
COPD: Chronic obstructive pulmonary disease
Covid-19: Coronavirus disease 2019
CoViDiab: Covid-19 & Diabetes study
CRP: C-reactive protein
DPP4i: Dipeptidyl peptidase 4 inhibitors
ESR: Erythrocyte sedimentation rate
EuGIA: Euglycaemic agents;
GLP-1RA: Glucagon-like peptide-1 receptor agonist
ICU: Intensive care unit
MDI: Multiple daily insulin injections
OHA: Oral hypoglycaemic agents
RAAS: Renin–angiotensin–aldosterone system
SGLT2i: Sodium-glucose co-transporter 2 inhibitors

## References

[1] Myers LC, Parodi SM, Escobar GJ, Liu VX (2020). Characteristics of Hospitalized Adults With COVID-19 in an Integrated Health Care System in California. JAMA..

[2] Goyal P, Choi JJ, Pinheiro LC, Schenck EJ, Chen R, Jabri A (2020). Clinical Characteristics of Covid-19 in New York City. N Engl J Med..

[3] Guan W, Ni Z, Hu Y, Liang W, Ou C, He J (2020). Clinical Characteristics of Coronavirus Disease 2019 in China. N Engl J Med..

[4] Richardson S, Hirsch JS, Narasimhan M, Crawford JM, McGinn T, Davidson KW (2020). Presenting characteristics, comorbidities, and outcomes among 5700 patients hospitalized With COVID-19 in the New York City Area. JAMA.

[5] Grasselli G, Zangrillo A, Zanella A, Antonelli M, Cabrini L, Castelli A (2020). Baseline Characteristics and Outcomes of 1591 Patients Infected with SARS-CoV-2 Admitted to ICUs of the Lombardy Region Italy.. J Am Med Assoc..

[6] Sardu C, Gargiulo G, Esposito G, Paolisso G, Marfella R (2020). Impact of diabetes mellitus on clinical outcomes in patients affected by Covid-19. Cardiovasc Diabetol.

[7] Fang L, Karakiulakis G, Roth M (2020). Are patients with hypertension and diabetes mellitus at increased risk for COVID-19 infection?. Lancet Respir Med.

[8] Maddaloni E, Buzzetti R (2020). Covid-19 and diabetes mellitus: unveiling the interaction of two pandemics. Diabetes. Metab. Res. Rev..

[9] Sardu C, D’Onofrio N, Balestrieri ML, Barbieri M, Rizzo MR, Messina V (2020). Outcomes in Patients With Hyperglycemia Affected by Covid-19: Can We Do More on Glycemic Control?. Diabetes Care..

[10] Sardu C, D’Onofrio N, Balestrieri ML, Barbieri M, Rizzo MR, Messina V (2020). Hyperglycaemia on admission to hospital and COVID-19. Diabetologia..

[11] Marfella R, Paolisso P, Sardu C, Bergamaschi L, D’Angelo EC, Barbieri M (2020). Negative impact of hyperglycaemia on tocilizumab therapy in Covid-19 patients. Diabetes Metab..

[12] Shi Q, Zhang X, Jiang F, Zhang X, Hu N, Bimu C (2020). Clinical Characteristics and Risk Factors for Mortality of COVID-19 Patients With Diabetes in Wuhan, China: A Two-Center. Retrospective Study. Diabetes Care..

[13] Haug N, Deischinger C, Gyimesi M, Kautzky-Willer A, Thurner S, Klimek P (2020). High-risk multimorbidity patterns on the road to cardiovascular mortality. BMC Med.

[14] Zhang D, Tang X, Shen P, Si Y, Liu X, Xu Z (2019). Multimorbidity of cardiometabolic diseases: prevalence and risk for mortality from one million Chinese adults in a longitudinal cohort study. BMJ Open.

[15] Glynn LG (2009). Multimorbidity: another key issue for cardiovascular medicine. Lancet.

[16] Luque-Fernandez MA, Schomaker M, Redondo-Sanchez D, Perez M, Vaidya A, Schnitzer ME (2019). Educational Note: Paradoxical collider effect in the analysis of non-communicable disease epidemiological data: a reproducible illustration and web application. Int J Epidemiol..

[17] Griffith G, Morris TT, Tudball M, Herbert A, Mancano G, Pike L, et al. Collider bias undermines our understanding of COVID-19 disease risk and severity. medRxiv. Cold Spring Harbor Laboratory Press; 2020. 10.1038/s41467-020-19478-2 PMC7665028 33184277

[18] Riddle MC, Buse JB, Franks PW, Knowler WC, Ratner RE, Selvin E (2020). COVID-19 in people with diabetes: urgently needed lessons from early reports. Diabetes Care..

[19] Guo W, Li M, Dong Y, Zhou H, Zhang Z, Tian C (2020). Diabetes is a risk factor for the progression and prognosis of COVID-19. Diabetes. Metab. Res. Rev..

[20] Zhu L, She Z-G, Cheng X, Qin J-J, Zhang X-J, Cai J (2020). Association of Blood Glucose Control and Outcomes in Patients with COVID-19 and Pre-existing Type 2 Diabetes. Cell Metab..

[21] Maddaloni E, D’Onofrio L, Pozzilli P (2016). Frailty and geography: should these two factors be added to the ABCDE contemporary guide to diabetes therapy?. Diabetes Metab Res Rev.

[22] Mathur R, Hull SA, Badrick E, Robson J (2011). Cardiovascular multimorbidity: the effect of ethnicity on prevalence and risk factor management. Br J Gen Pract.

[23] Maddaloni E, D'Onofrio L, Alessandri F, Mignogna C, Leto G, Coraggio L (2020). Clinical features of patients with type 2 diabetes with and without Covid-19: a case control study (CoViDiab I). Diabetes Res Clin Pract.

[24] World Health Organization. Laboratory testing for coronavirus disease 2019 (COVID-19) in suspected human cases.

[25] Sardu C, Maggi P, Messina V, Iuliano P, Sardu A, Iovinella V (2020). Could Anti-Hypertensive Drug Therapy Affect the Clinical Prognosis of Hypertensive Patients With COVID-19 Infection? Data From Centers of Southern Italy. J. Am. Heart Assoc..

[26] Strollo R, Pozzilli P (2020). DPP4 inhibition: Preventing SARS-CoV-2 infection and/or progression of COVID-19?. Diabetes. Metab Res Rev..

[27] Guan W, Liang W, Zhao Y, Liang H, Chen Z, Li Y (2020). Comorbidity and its impact on 1590 patients with Covid-19 in China: A Nationwide Analysis. Eur. Respir. J..

[28] Banerjee A, Pasea L, Harris S, Gonzalez-Izquierdo A, Torralbo A, Shallcross L (2020). Estimating excess 1-year mortality associated with the COVID-19 pandemic according to underlying conditions and age: a population-based cohort study. Lancet.

[29] Pascarella G, Strumia A, Piliego C, Bruno F, Del Buono R, Costa F (2020). COVID-19 diagnosis and management: a comprehensive review. J. Intern. Med..

[30] Ackermann M, Verleden SE, Kuehnel M, Haverich A, Welte T, Laenger F (2020). Pulmonary vascular endothelialitis, thrombosis, and angiogenesis in Covid-19. N. Engl. J. Med..

[31] Madjid M, Safavi-Naeini P, Solomon SD, Vardeny O (2020). Potential effects of coronaviruses on the cardiovascular system. JAMA Cardiol..

[32] Sardu C, Gambardella J, Morelli MB, Wang X, Marfella R, Santulli G (2020). Hypertension, thrombosis, kidney failure, and diabetes: is COVID-19 an endothelial disease? A comprehensive evaluation of clinical and basic evidence. J Clin Med.

[33] Varga Z, Flammer AJ, Steiger P, Haberecker M, Andermatt R, Zinkernagel AS (2020). Endothelial cell infection and endotheliitis in COVID-19. Lancet.

[34] Rask-Madsen C, King GL (2013). Vascular Complications of Diabetes: Mechanisms of Injury and Protective Factors. Cell Metab..

[35] Patti G, Cavallari I, Andreotti F, Calabrò P, Cirillo P, Denas G (2019). Prevention of atherothrombotic events in patients with diabetes mellitus: from antithrombotic therapies to new-generation glucose-lowering drugs. Nat Rev Cardiol.

[36] Panigada M, Bottino N, Tagliabue P, Grasselli G, Novembrino C, Chantarangkul V (2020). Hypercoagulability of COVID-19 patients in Intensive Care Unit. A report of thromboelastography findings and other parameters of hemostasis. J. Thromb. Haemost..

[37] Leisman DE, Deutschman CS, Legrand M (2020). Facing COVID-19 in the ICU: vascular dysfunction, thrombosis, and dysregulated inflammation. Intensive Care Med..

[38] Petersen A, Bressem K, Albrecht J, Thieß H-M, Vahldiek J, Hamm B (2020). The role of visceral adiposity in the severity of COVID-19: Highlights from a unicenter cross-sectional pilot study in Germany. Metabolism.

[39] Zheng KI, Gao F, Wang X-B, Sun Q-F, Pan K-H, Wang T-Y (2020). Letter to the Editor: Obesity as a risk factor for greater severity of COVID-19 in patients with metabolic associated fatty liver disease. Metabolism.

[40] da Silva A, Caldas APS, Hermsdorff HHM, Bersch-Ferreira ÂC, Torreglosa CR, Weber B (2019). Triglyceride-glucose index is associated with symptomatic coronary artery disease in patients in secondary care. Cardiovasc Diabetol.

[41] Cull CA, Jensen CC, Retnakaran R, Holman RR (2007). Impact of the metabolic syndrome on macrovascular and microvascular outcomes in type 2 diabetes mellitus. Circulation.

[42] Sandoval Y, Januzzi JL, Jaffe AS (2020). Cardiac Troponin for the Diagnosis and Risk-Stratification of Myocardial Injury in COVID-19: JACC Review Topic of the Week.

[43] Oluwagbemigun K, Buyken AE, Alexy U, Schmid M, Herder C, Nöthlings U (2019). Developmental trajectories of body mass index from childhood into late adolescence and subsequent late adolescence-young adulthood cardiometabolic risk markers. Cardiovasc Diabetol.

