# Supplementary material for: Cardiometabolic multimorbidity is associated with a worse Covid-19 prognosis than individual cardiometabolic risk factors: a multicentre retrospective study (CoViDiab II)
Source: Cardiovasc Diabetol. 2020 Oct 1;19:164. doi: 10.1186/s12933-020-01140-2 (PMC7528157; doi:10.1186/s12933-020-01140-2)
Supplement: Supplementary file 1 — Additional file 1:Table S1. SARS-CoV-2 infection symptoms at hospitalization in patients with, compared with those without, diabetes and in patients experiencing the primary composite compared with those without. Table S2. Odds ratio (OR) with [95% confidence intervals, CI] for secondary outcomes, unadjusted and adjusted for age and sex. Abbreviations: CV, cardiovascular; COPD, chronic obstructive pulmonary disease. [file 12933_2020_1140_MOESM1_ESM.docx]

**Additional file** to *Maddaloni E et al, Cardiometabolic multimorbidity is associated with a worsen Covid-19 prognosis than single cardiometabolic risk factors: a multicentre retrospective study (CoViDiab II).*

**Additional file 1:Table S1**. SARS-CoV-2 infection symptoms at hospitalization in patients with, compared with those without, diabetes and in patients experiencing the primary composite compared with those without.

|  | **Diabetes** | |  | **Primary Composite Outcome** | |  |
| --- | --- | --- | --- | --- | --- | --- |
|  | **No (n=273)** | **Yes (n=81)** | **p** | **No (n=149)** | **Yes (n=128)** | **p** |
| Fever n | 228 (83.8%) | 63 (77.8%) | 0.21 | 125 (83.3%) | 102 (78.5%) | 0.30 |
| Cold n | 9 (5.0%) | 2 (3.3%) | 0.58 | 6 (5.0%) | 5 (5.1%) | 0.98 |
| Conjunctivitis n | 9 (3.4%) | 1 (1.3%) | 0.35 | 6 (4.0%) | 2 (1.6%) | 0.24 |
| Chest pain n | 18 (6.7%) | 6 (7.9%) | 0.73 | 12 (8.1%) | 7 (5.8%) | 0.47 |
| Dyspnea n | 140 (51.7%) | 36 (46.1%) | 0.39 | 61 (40.9%) | 84 (66.1%) | <0.001 |
| Nausea or vomiting n | 8 (3.0%) | 4 (5.2%) | 0.36 | 4 (2.7%) | 6 (4.9%) | 0.34 |
| Diarrhoea n | 24 (9.0%) | 11 (14.5%) | 0.17 | 16 (10.7%) | 9 (7.6%) | 0.38 |
| Anosmia n/n_obs | 9/180 (5.0%) | 2/61 (3.3%) | 0.58 | 9/119 (7.6%) | 2/97 (2.1%) | 0.067 |
| Ageusia n/n_obs | 9/180 (5.0%) | 1/61 (1.6%) | 0.26 | 9/119 (7.6%) | 1/97 (1.0%) | 0.023 |

**Additional file 1: Table S2.** Odds ratio (OR) with [95% confidence intervals, CI] for secondary outcomes, unadjusted and adjusted for age and sex. Abbreviations: CV, cardiovascular; COPD, chronic obstructive pulmonary disease;

|  | **ICU admission or death** | | | | **Died** | | | |
| --- | --- | --- | --- | --- | --- | --- | --- | --- |
|  | **No** | | **Yes** | | **No** | | **Yes** | |
|  | **n=182** | | **n=77** | | **n=199** | | **n=29** | |
|  | **Unadjusted** | | **Age- and sex-adjusted** | | **Unadjusted** | | **Age- and sex-adjusted** | |
|  | **OR**  **[95%CI]** | **p** | **OR**  **[95%CI]** | **p** | **OR**  **[95%CI]** | **p** | **OR**  **[95%CI]** | **p** |
| **Age ≥70 years** | 3.52  [2.02–6.13] | <0.001 | N/A | | 5.15  [2.22–11-96] | <0.001 | N/A | |
| **Male sex** | 1.51  [0.86–2.66] | 0.15 | N/A | | 0.89  [0.41–1.97] | 0.78 | N/A | |
| **Diabetes** | 1.84  [1.00–3.36] | 0.049 | 1.39  [0.73–2.64] | 0.31 | 1.85  [0.78–4.37] | 0.16 | 1.25  [0.50–3.13] | 0.63 |
| **Prior hypertension** | 2.54  [1.45–4.44] | 0.001 | 1.81  [0.99–3.31] | 0.052 | 4.37  [1.71–11.19] | 0.002 | **2.75**  **[1.01–7.49]** | **0.048** |
| **Prior COPD** | 5.73  [2.78–11.84] | <0.001 | **4.22**  **[1.97–9.03]** | **<0.001** | 6.51  [2.65–16.00] | <0.001 | **4.63**  **[1.77–12.10]** | **0.002** |
| **Dyslipidaemia** | 1.81  [0.98–3.34] | 0.058 | 1.47  [0.77–2.81] | 0.22 | 1.88  [0.79–4.46] | 0.15 | 1.34  [0.54–3.32] | 0.53 |
| **Prior CV event** | 2.29  [1.06–4.97] | 0.036 | 1.42  [0.62–3.26] | 0.41 | 3.18  [1.20–8.47] | 0.020 | 1.99  [0.69–5.72] | 0.20 |
| **Prior heart failure** | 5.71  [2.05–15.86] | 0.001 | **3.53**  **[1.21–10.30]** | **0.021** | 7.36  [2.27–23.88] | 0.001 | **4.23**  **[1.21–14.77]** | **0.024** |
| **Prior malignancy** | 1.97  [0.71–5.49] | 0.20 | 1.60  [0.54–4.73] | 0.39 | 2.16  [0.56–8.36] | 0.27 | 1.49  [0.36–6.15] | 0.59 |
| **Smoking (ever)** | 0.97  [0.49–1.93] | 0.93 | 0.75  [0.36–1.57] | 0.45 | 1.35  [0.53–3.45] | 0.53 | 0.97  [0.35–2.70] | 0.96 |
